# New and Interesting Taxa from the Diatom Genus *Gomphonema* Ehrenberg in Shallow, Nearshore Sites on the Eastern Coast of Lake Baikal

**DOI:** 10.3390/plants12091835

**Published:** 2023-04-29

**Authors:** Maxim S. Kulikovskiy, John Patrick Kociolek, Cüneyt Nadir Solak, Irina V. Kuznetsova, Anton M. Glushchenko

**Affiliations:** 1 K.A. Timiryazev Institute of Plant Physiology RAS, IPP RAS, 35 Botanicheskaya St., 127276 Moscow, Russia; 2 Museum of Natural History, Henderson Building, 15th and Broadway, Boulder, CO 80309, USA; 3 Department of Biology, Art & Science Faculty, Dumlupınar University, Kütahya 43100, Turkey

**Keywords:** Bacillariophyceae, new species, *Gomphonema*, morphology, systematics, taxonomy, Lake Baikal

## Abstract

During this investigation, sixteen species from the genus *Gomphonema* were found in a shallow bay (near Oimur, Kabansky District) located on the eastern shore of Lake Baikal. Eleven of these taxa have been described previously. Five species are described as new to science. We provide ecological information about these *Gomphonema* species, including their distribution within and outside Lake Baikal, and compare and contrast the new taxa with morphologically similar congeners. The diversity of morphologies present in Lake Baikal among the *Gomphonema* species suggests there has been both in-lake speciation as well as the introduction of various groups of species. The idea that Lake Baikal may support a high number of endemics by their partitioning niches based on depth is supported by the different groups of gomphonemoid diatoms present.

## 1. Introduction

Lake Baikal is a unique aquatic ecosystem, being the oldest continuously existing water body [1] and supporting high levels of endemic species across many groups of organisms [2,3,4,5]. A comprehensive picture of the diversity of freshwater diatoms in Lake Baikal is coming into focus, based on earlier studies that proposed about 400 new taxa [6,7,8,9,10,11,12,13,14,15], more recent works published in two monographs, and many manuscripts that have revealed 13 new genera and more than an additional 400 new species [16,17,18,19,20,21,22,23,24,25,26,27,28,29,30,31,32,33,34,35,36,37,38,39,40,41].

In early revisions of the diatoms from Lake Baikal, Skvortzow and Meyer [13], Skvortzow [14], and Meyer [12] reported about 40 specific and infraspecific taxa from the genus *Gomphonema* Ehrenberg. Some of the discussed taxa belong to the genus *Gomphoneis* Cleve (=*Gomphonella* Rabenhorst nowadays) [33,42]. About twenty specific and infraspecific taxa were described as new to science and some of them were considered as endemic diatoms [13,14]. The history of diatom research from Lake Baikal was discussed by us previously [20]. Three more recent publications were dedicated to documenting the genus *Gomphonema* [25,34,43] in Lake Baikal. The morphology and distribution of endemic and widespread taxa from Lake Baikal were presented. Comprehensive light microscopic documentation of the valve diminution series of *Gomphonema* taxa is a basis for our understanding of species diversity in ancient Lake Baikal and the biogeography of the taxa present there. For example, a previous investigation of taxa from the *G. acuminatum* and *G*. *truncatum–capitatum* species complexes in Lake Baikal revealed some new species for science [25]. Some of the taxa treated in that publication were previously observed from other regions, especially from Mongolia. Our data show a wide distribution of species in the waterbodies of Central Asia and confirm the presence of some cosmopolitan taxa within Lake Baikal [25]. Some publications were dedicated to the discussion of *Gomphonema* species composition in the Baikal region, which are additional resources for taxonomical reinvestigation of diatom taxa [44].

Lake Baikal is the deepest lake in the world, being more than 1500 m in depth. The theory of “immiscibility” suggests there are endemic species found only in Baikal (and not in the surrounding lotic and lentic systems), while other species are found only in the surrounding aquatic habitats. There are many papers written about this phenomenon in the Russian literature [45], especially about the lake’s fauna. For diatoms, the plankton flora may not conform to this theory [46,47] due to the relatively recent development of the current species composition, while data on the benthic flora seem to support this idea.

The aim of this study is a taxonomic review and documentation of the species diversity of the *Gomphonema* taxa from a shallow water bay located in an underexplored region in the southern part of Lake Baikal. We compare the distribution of the species considered herein with others from deep waters.

## 2. Results and Discussion

During this investigation, 16 *Gomphonema* species were found and, of them, five are new to science. The previously-known taxa include *Gomphonema makarovae* Lange-Bertalot in Lange-Bertalot and Genkal 1999, *G. distans* (Cleve-Euler) Lange-Bertalot and Reichardt in Lange-Bertalot and Genkal 1999, *G. subarcticum* Lange-Bertalot and Reichardt in Lange-Bertalot and Genkal 1999, *G. parvulius* (Lange-Bertalot and Reichardt) Lange-Bertalot and Reichardt in Lange-Bertalot and Metzeltin 1996, *G. duplipunctatum* Lange-Bertalot and Reichardt in Lange-Bertalot, Kulbs, Lauser, Norpel-Schempp and Willmann 1996, *G. sphenovertex* Lange-Bertalot and Reichardt in Lange-Bertalot and Metzeltin 1996, *G. jergackianum* Reichardt 2009, *G. popovae* Levadnaja emend. Vishnjakov and Romanov 2015, *G. medioasiae* Metzeltin, Lange-Bertalot and Nergui 2009, *G. demersum* Reichardt 2009 and *G. pseudoaugur* Lange-Bertalot 1979. Descriptions of new species follow the presentation of the previously described taxa.

**Figure 1:**
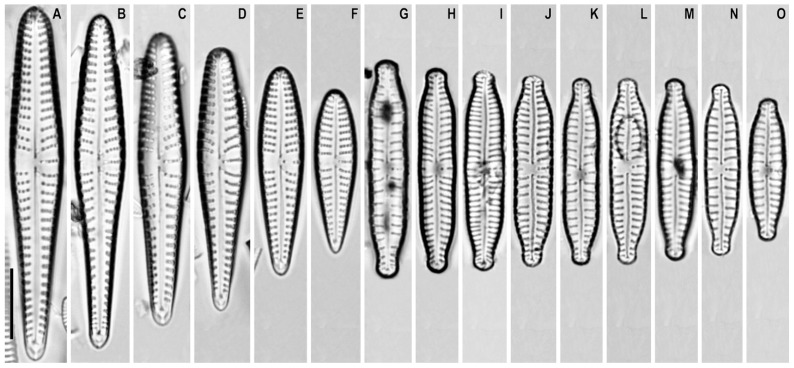
(**A**–**O**). *Gomphonema* spp. Light microscopy, differential interference contrast, size diminution series. (**A**–**F**). *G. makarovae*. Slide no. 18589. (**G**–**O**). *G. subarcticum*. Slide no. 18599. Scale bar = 10 μm.

*Gomphonema makarovae* Lange-Bertalot in Lange-Bertalot and Genkal (Figure 1A–F)

**Remarks.** The specimens in our samples were 23.5–50.0 μm long, 6.0–7.5 μm wide, and had 9–11 striae in 10 μm.

This species was found on slide no. 18589.

*Gomphonema subarcticum* Lange-Bertalot and Reichardt in Lange-Bertalot and Genkal (Figure 1G–O)

**Synonym:** *Gomphonema angustatum* var. *undulatum* Grunow sensu auct. nonnull.

**Remarks.** The specimens in our samples were 20.0–31.0 μm long, 5.0–5.8 μm wide, and had 8–12 striae in 10 μm.

This species was found on slide no. 18599.

**Figure 2:**
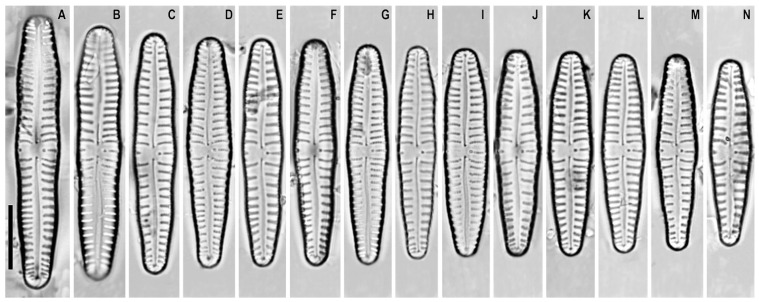
(**A**–**N**). *Gomphonema distans*. Light microscopy, differential interference contrast, size diminution series. Slide no. 18606. Scale bar = 10 μm.

*Gomphonema distans* (Cleve-Euler) Lange-Bertalot and Reichardt in Lange-Bertalot and Genkal (Figure 2A–N)

**Basionym:** *Gomphonema lagerheimii* var. *distans* A. Cleve 1934.

**Remarks.** The specimens in our samples were 29.0–42.5 μm long, 6.0–7.0 μm wide, and had 8–11 striae in 10 μm.

This species was found on slide no. 18606.

**Figure 3:**
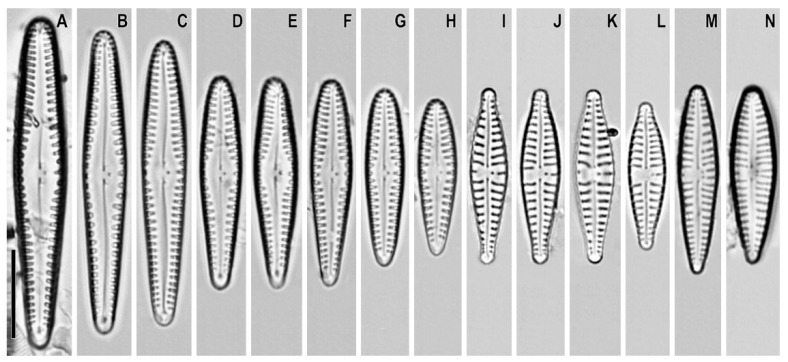
(**A**–**N**). *Gomphonema* spp. Light microscopy, differential interference contrast, size diminution series. (**A**–**H**). *G. demersum*. Slides no. 18589 and 18606. (**I**–**L**). *G. parvulius*. Slides no. 18589 and 18599. (**M**,**N**). *G. duplipunctatum*. Slides no. 18599 and 18606. Scale bar = 10 μm.

*Gomphonema demersum* Reichardt 2009 (Figure 3A–H)

**Remarks.** The specimens in our samples were 18–38 μm long, 4.5–6.0 μm wide, and had 12–14 striae in 10 μm.

This species was found on slides no. 18589 and 18606.

*Gomphonema parvulius* (Lange-Bertalot and Reichardt) Lange-Bertalot and Reichardt in Lange-Bertalot and Metzeltin (Figure 3I–L)

**Basionym:** *Gomphonema parvulum* var. *parvulius* Lange-Bertalot and Reichardt in Lange-Bertalot 1993

**Remarks.** The specimens in our samples were 17.0–20.0 μm long, 4.5–5.9 μm wide, and had 12–14 striae in 10 μm.

This species was found in slides no. 18589 and 18599.

*Gomphonema duplipunctatum* Lange-Bertalot et Reichardt in Lange-Bertalot, Kulbs, Lauser, Norpel-Schempp and Willmann (Figure 3M,N)

**Synonym:** *Gomphonema bipunctatum* Krasske 1943.

**Remarks.** The specimens in our samples were 20.0–21.5 μm long, 4.0–4.5 μm wide, and had 12–14 striae in 10 μm.

This species was found on slides no. 18599 and 18606.

**Figure 4:**
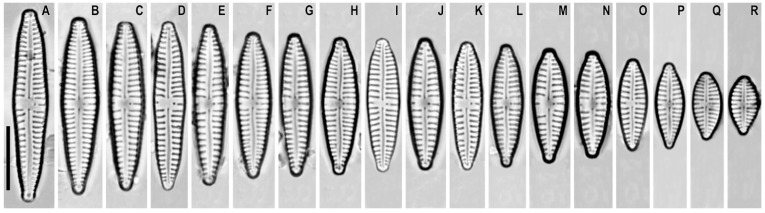
(**A**–**R**). *Gomphonema sphenovertex*. Light microscopy, differential interference contrast, size diminution series. Slide no. 18606. Scale bar = 10 μm.

*Gomphonema sphenovertex* Lange-Bertalot et Reichardt in Lange-Bertalot and Metzeltin (Figure 4A–R)

**Remarks.** The specimens in our samples were 9.5–30.5 μm long, 4.5–6.9 μm wide, and had 12–16 striae in 10 μm.

This species was found on slide no. 18606.

**Figure 5:**
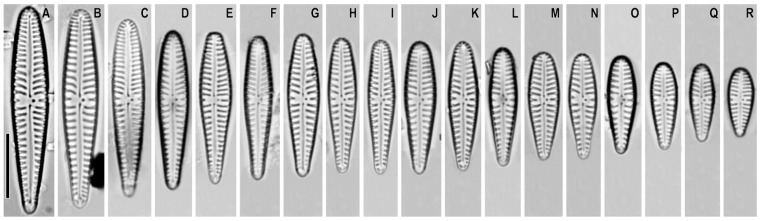
(**A**–**R**). *Gomphonema jergackianum*. Light microscopy, differential interference contrast, size diminution series. Slides no. 18589 and 18599. Scale bar = 10 μm.

*Gomphonema jergackianum* Reichardt (Figure 5A–R)

**Remarks.** The specimens in our samples were 11.0–32.5 μm long, 4.0–6.0 μm wide, and had 11–14 striae in 10 μm.

This species was found on slides no. 18589 and 18599.

**Figure 6:**
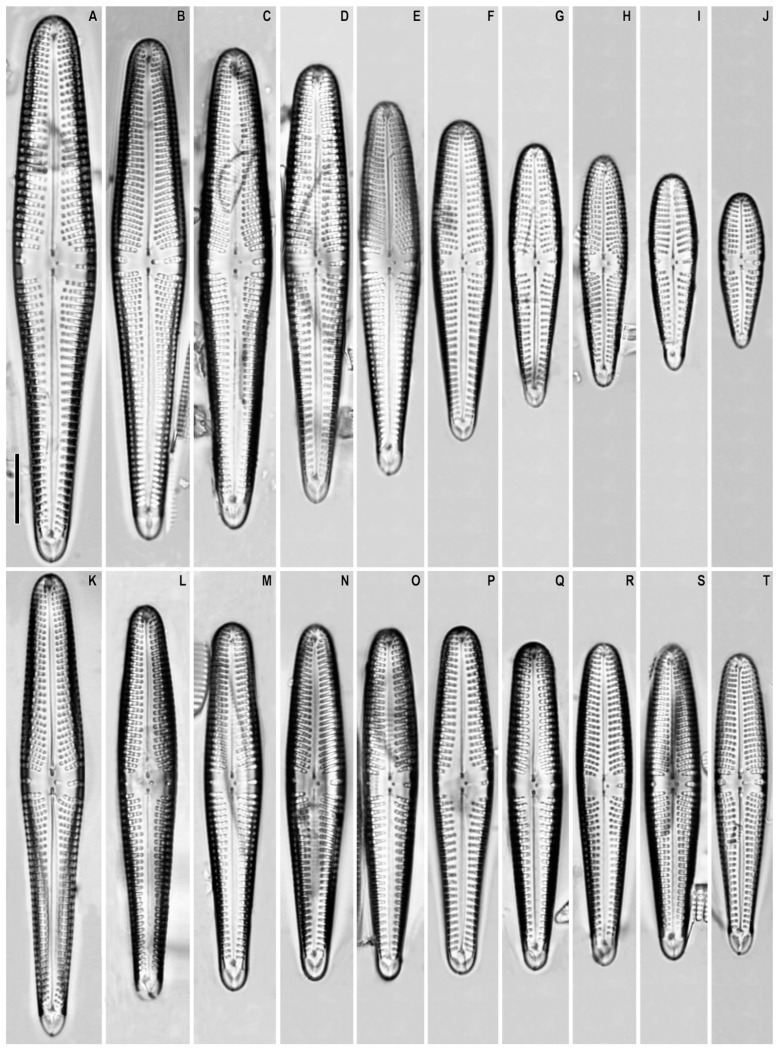
(**A**–**T**). *Gomphonema popovae*. Light microscopy, differential interference contrast, size diminution series. Slide no. 18589. Scale bar = 10 μm.

*Gomphonema popovae* Levadnaja emend. Vishnjakov and Romanov (Figure 6A–T)

**Synonym:** *Gomphonema liyanlingae* Metzeltin and Lange-Bertalot in Metzeltin et al. 2009

**Remarks.** The specimens in our samples were 22.5–78.0 μm long, 6.8–12.0 μm wide, and had 10–13 striae in 10 μm.

This species was found on slide no. 18599.

**Comments.** This is an oligotrophic species [48,49]. Our specimens have expanded striae density as compared to previous reports (Table 1). It is a highly variable taxon, as previously discussed [48]. In one sample, we found two morphotypes of this species. The first morphotype (Figure 6A–J; Table 1) has wider valves that are more club shaped. The second morphotype (Figure 6K–J; Table 1) has narrower valves, less widened in their central part. At the same time, the densities of striae and areolae are identical for these two morphotypes (see Table 1). Our specimens are somewhat different from both the material shown by Vishnyakov and Romanov [48] and from the material shown from Mongolia [50] (Table 1). At the same time, no noticeable morphological differences were found to separate these populations.

**Figure 7:**
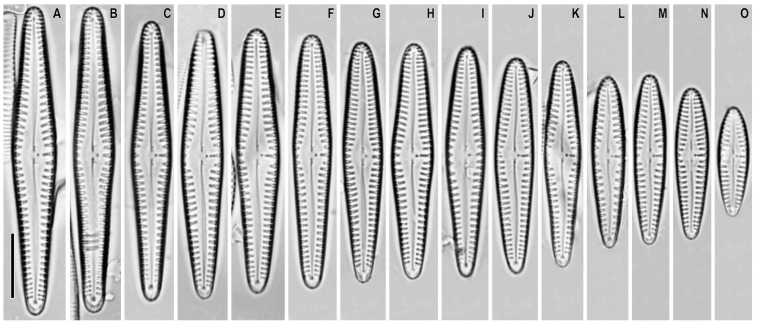
(**A**–**O**). *Gomphonema medioasiae*. Light microscopy, differential interference contrast, size diminution series. Slide no. 18599. Scale bar = 10 μm.

*Gomphonema medioasiae* Metzeltin, Lange-Bertalot and Nergui (Figure 7A–O)

**Remarks.** The specimens in our samples were 17.0–48.0 μm long, 4.5–7 μm wide, and had 10–13 striae in 10 μm.

This species was found on slide no. 18599.

**Figure 8:**
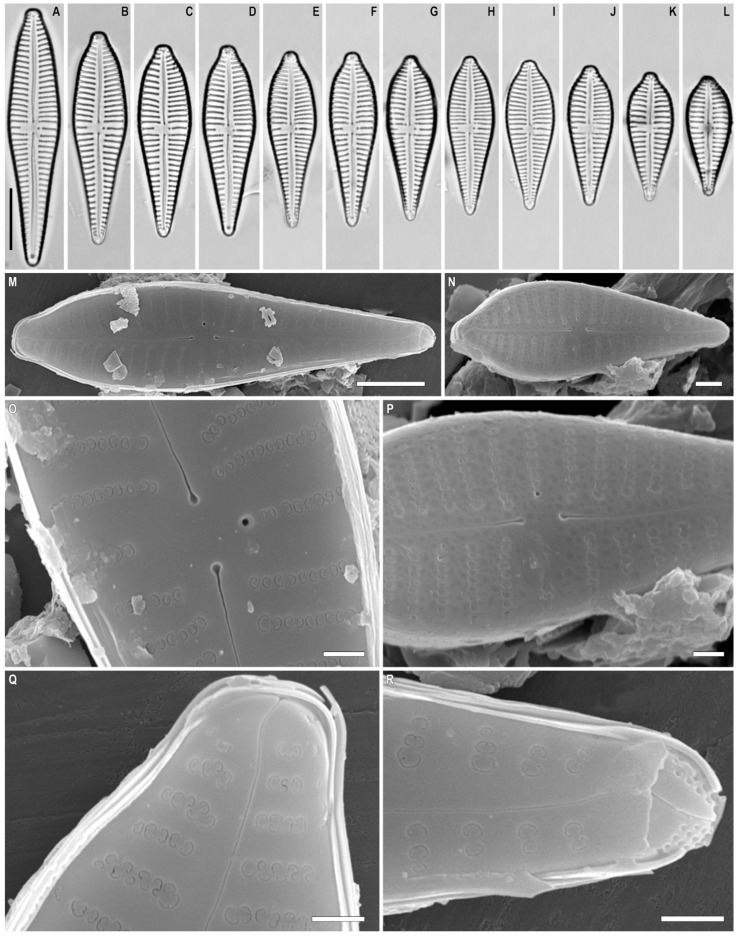
(**A**–**R**). *Gomphonema pseudoaugur* Lange-Bertalot (**A**–**L**). Light microscopy, differential interference contrast, size diminution series. Slide no. 18599. (**M**–**P**). Scanning electron microscopy, external views. Scale bar (**A**–**L**) = 10 μm; (**M**) = 5 μm; (**N**) = 2 μm; (**O**–**R**) = 1 μm.

**Figure 9:**
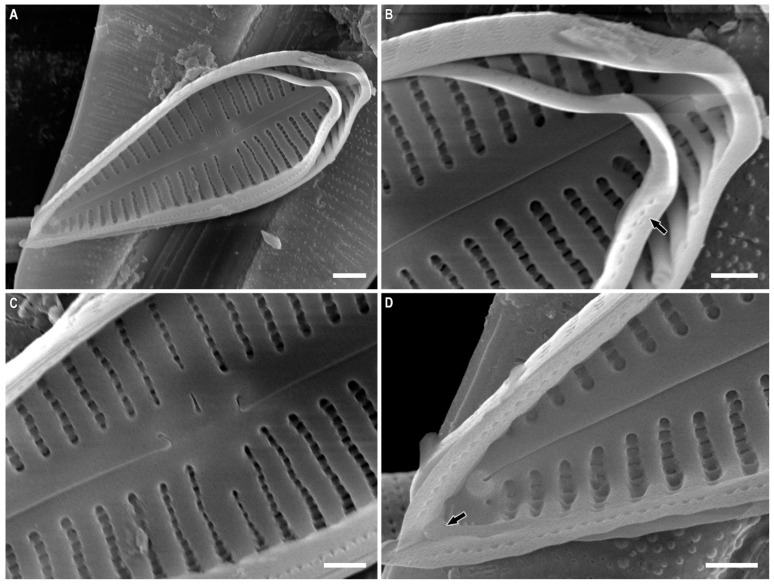
(**A**–**D**). *Gomphonema pseudoaugur* Lange-Bertalot. Scanning electron microscopy, internal views. (**B**). The black arrow shows the septum. (**D**). The black arrow shows the pseudoseptum. Scale bar (**A**) = 2 μm; (**B**–**D**) = 1 μm.

*Gomphonema pseudoaugur* Lange-Bertalot (Figure 8A–R and Figure 9A–D)

**Description.** LM (Figure 8A–L). Valves are distinctly heteropolar, clavate with broadly rounded and short rostrate headpole, and acute footpole. The valve length is 19.5–42.0 µm, breadth 7.0–9.0 µm. The axial area is narrow and linear. The central area is small, transversally elongated made by the shortening of one central stria. Central striae on both valve sides are distantly spaced from the other striae. One isolated pore is present in the central area located very close to the slightly shortened central stria. Striae fine and uniseriate, radiate, 12–16 in 10 µm.

SEM, external view (Figure 8M–R). Striae uniseriate. The areolae are occluded by flaps that create *c*-shaped openings, ≈40 in 10 µm. The proximal raphe endings are expanded, pore-like, and deflected towards the isolated round pore. The apical pore field is composed of round porelli.

SEM, internal view (Figure 9A–D). The areolae are located in narrow foraminal rows between strongly silificed vimines. Beneath the vimines, the sides of the grooves bear pairs of small struts. The proximal raphe ends are hooked and located on a raised central nodule. There is an isolated pore with a long slit-like opening. The septa are present (Figure 9B, black arrow). Small pseudosepta are present, visible at footpoles (Figure 9D, black arrow). The helictoglossae are offset from the raphe branch. A small septum is present at the headpole, though one has not been seen at the footpole.

**New species:**

**Figure 10:**
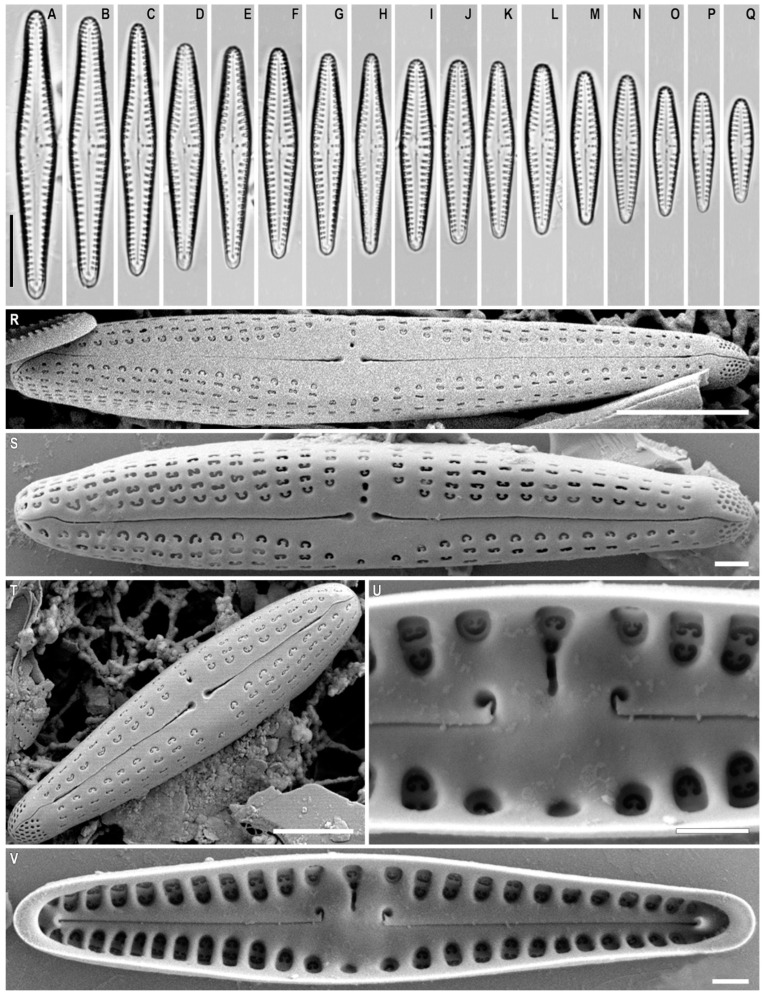
(**A**–**Q**). *Gomphonema baicalodemersum* Kulikovskiy, Kociolek, Solak and *Glushchenko* sp. nov. (**A**–**Q**). Light microscopy, differential interference contrast, size diminution series. Slide no. 18589. (**R**–**T**). Scanning electron microscopy, external views. (**U**,**V**). Scanning electron microscopy, internal views. (**D**). Holotype. Scale bar (**A**–**Q**) = 10 μm; (**S**) = 6 μm; (**T**) = 3 μm; (**S**,**U**,**V**) = 1 μm.

*Gomphonema baicalodemersum* Kulikovskiy, Kociolek, Solak and *Glushchenko* sp. nov. (Figure 10A–V)

**Holotype.** Deposited in the herbarium of MHA, Main Botanical Garden Russian Academy of Science, Moscow, Russia, the holotype here designated, slide no. 18589 (Figure 10D).

**Isotype.** Collection of Maxim Kulikovskiy at the Herbarium of the Institute of Plant Physiology Russian Academy of Science, Moscow, Russia, slide no. 18589a.

**Description.** LM (Figure 10A–Q). The valves are slightly heteropolar, rhombic lanceolate (in larger specimens), or very slightly clavate (in smaller specimens) with acutely rounded headpoles and footpoles. Valve length 14.5–38.5 µm and breadth 3–6 µm. Raphe filiform, weakly lateral with proximal ends dilated slightly and distal ends deflected onto the valve mantle. The axial area is lanceolate, with a widening at the central area. The central area is weakly expressed. There is an isolated pore near the central nodule and densely spaced to the rather long median stria. The striae are short and radiate, 11–15 in 10 µm.

SEM, external view (Figure 10R–T). The striae are uniseriate. The areolae are occluded by large reniform siliceous flaps and merged with the valve surface, ≈30 in 10 µm. The proximal raphe endings are pore-like and deflected towards the isolated round pore. There is a prominent bilobed apical pore field composed of round porelli and bisected by external distal-raphe ends.

SEM, internal view (Figure 10U,V). The areolar openings are large and round with reniform external occlusion located in a deep foraminal row between the strongly silicified vimines. There is an isolated pore that is large, transversely elongated, and located in a long groove. The proximal raphe ends are long, right-angled, terminating with a pore located on a thickened ridge. Pseudosepta are present and visible at both poles. The helictoglossae are offset from the raphe branch.

**Comments.** *Gomphonema baicalodemersum* sp. nov. belongs to the group of species with a wide axial area considered by Reichardt [51] and a number of other authors. Species of this group are known from Africa, Australasia, Europe, Asia, and North and South America [44,51,52,53]. A distinctive feature of *G. baicalodemersum* sp. nov. is the presence of a long internal slit-like opening of the isolated pore, which passes into the foraminal row. In the light microscope, this structure is perceived as an elongated stria. A similar isolated pore structure is present in *G. medioasiae* [50]. At the same time, *G. baicalodemersum* sp. nov. has a narrower headpole and footpole of valves than *G. medioasiae*. The valve width of *G. baicalodemersum* sp. nov. (3–6 µm) is mostly narrower than the width of *G. medioasiae* (4.5–8.0 µm).

*G. baicalodemersum* sp. nov. is similar to *G. demersum* (Table 2) and the two species have similar valve widths (3–6 µm in *G. baicalodemersum* sp. nov. versus 3.6–6.0 µm in *G. demersum*) and striae densities (11–16 in 10 µm in *G. baicalodemersum* sp. nov. versus 12–16 in 10 µm in *G. demersum*). The two differ in that *G. baicalodemersum* sp. nov. has a more narrow headpole as compared to *G. demersum*.

Additionally, the internal slit-like opening of the isolated pore of *G. baicalodemersum* sp. nov. is quite extended and connected with a narrow foraminal row of stria, while in *G. demersum* the isolated pore opening also has a slit-like shape, though less extended and does not reach the marginal stria.

**Figure 11:**
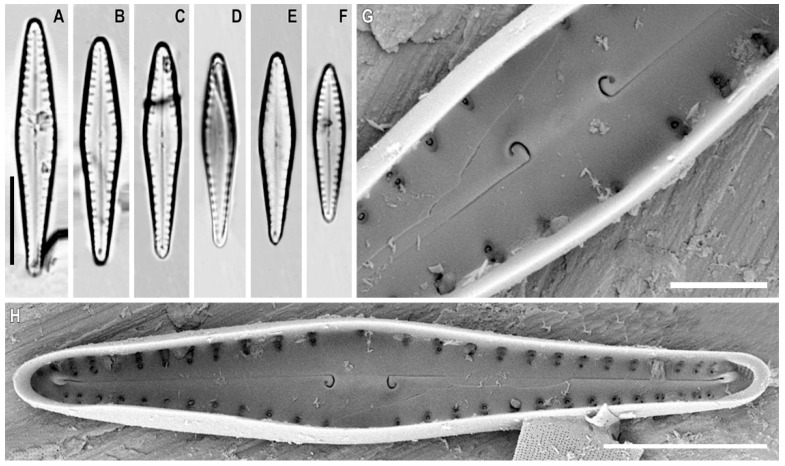
(**A**–**H**). *Gomphonema genkalii* Kulikovskiy, Kociolek, Solak and *Glushchenko* sp. nov. (**A**–**F**). Light microscopy, differential interference contrast, size diminution series. Slide no. 18607. (**G**,**H**). Scanning electron microscopy, internal views. (**B**). Holotype. Scale bar (**A**–**F**) = 10 μm; (**H**) = 6 μm; (**G**) = 2 μm.

*Gomphonema genkalii* Kulikovskiy, Kociolek, Solak and *Glushchenko* sp. nov. (Figure 11A–H)

**Holotype.** Deposited in the herbarium of MHA, Main Botanical Garden Russian Academy of Science, Moscow, Russia, holotype here designated, slide no. 18607 (Figure 11B).

**Isotype.** Collection of Maxim Kulikovskiy at the Herbarium of the Institute of Plant Physiology Russian Academy of Science, Moscow, Russia, slide no. 18607a.

**Type locality.** Russia, Lake Baikal, shallow-water bay, *Cladophora* spp. On the bottom, 52°27.042′ N; 106°53.215′ E. Collected by M.S. Kulikovskiy, 14 July 2014, pH = 9.3, conductivity = 151 µS cm^−1^, t = 21.3 °C.

**Etymology.** Species dedicated to the Russian diatomist Dr. Sergei Genkal.

**Distribution.** Known only from the type locality.

**Description.** LM (Figure 11A–F). The valves are heteropolar, rhombic lanceolate (in larger specimens), or very slightly clavate (in smaller specimens) with acutely rounded headpoles and footpoles. The valve length is 18.0–29.5 µm and the breadth is 4.0–4.5 µm. The raphe is straight with proximal ends dilated slightly and distal ends deflected onto the valve mantle. The axial area is narrow but expands near the central area. Isolated pores are absent. The striae are short and weakly radiate, 12–14 in 10 µm.

SEM, internal view (Figure 11G,H). The areolar openings are small, round, and located in shallow foraminal rows. The side walls of the areolae bear part of small struts. The proximal raphe ends are hooked and located on a raised central nodule. The pseudosepta is poorly defined and visible at both poles. The helictoglossae is offset from the raphe branch.

**Comments.** *Gomphonema genkalii* sp. nov. can be assigned to the group of species with a wide axial area considered by Reichardt [51]. At the same time, the species has a unique valve outline and an absent isolated pore that makes it difficult to compare with any species of this group.

**Figure 12:**
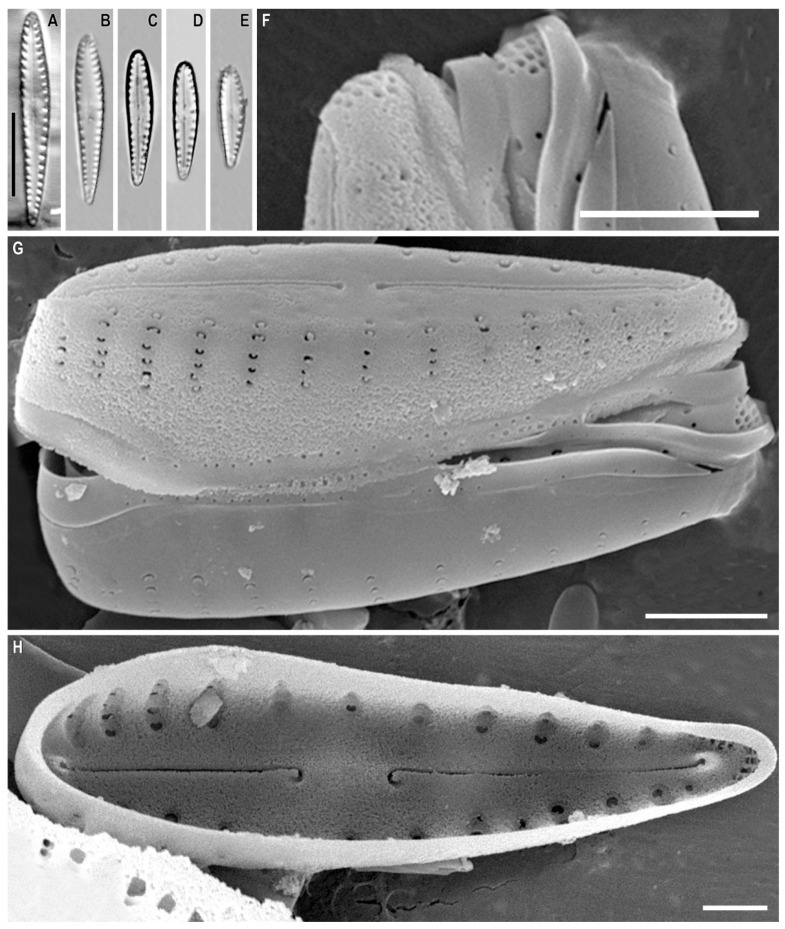
(**A**–**H**). *Gomphonema trifonovae* Kulikovskiy, Kociolek, Solak and *Glushchenko* sp. nov. (**A**–**E**). Light microscopy, differential interference contrast, size diminution series. Slide no. 18608. (**F**,**G**). Scanning electron microscopy, external views. (**H**). Scanning electron microscopy, internal view. (**C**). Holotype. Scale bar (**A**–**E**) = 10 μm; (**F**,**G**) = 1 μm; (**H**) = 1 μm.

*Gomphonema trifonovae* Kulikovskiy, Kociolek, Solak and *Glushchenko* sp. nov. (Figure 12A–H)

**Holotype.** Deposited in the herbarium of MHA, Main Botanical Garden Russian Academy of Science, Moscow, Russia, holotype here designated, slide no. 18608 (Figure 12C).

**Isotype.** Collection of Maxim Kulikovskiy at the Herbarium of the Institute of Plant Physiology Russian Academy of Science, Moscow, Russia, slide no. 18608a.

**Type locality.** Russia, Lake Baikal, shallow-water bay, bottom sediments, and detritus in a clump of *Carex* spp., 52°27.042′ N; 106°53.215′ E. Collected by M.S. Kulikovskiy, 14 July 2011, pH = 9.0, conductivity = 456 µS cm^−1^, t = 18.2 °C.

**Etymology.** This species is dedicated to the Russian hydrobiologist, Prof. Dr. Irina Trifonova from the Institute of Limnology, Saint Petersburg.

**Distribution.** Known only from the type locality.

**Description.** LM (Figure 12A–E). The valves are clavate and are asymmetrical to the longitudinal axis with an acute to narrow rounded headpole. The footpole is acutely rounded. The valve length is 13–26 µm and the breadth is 2.8–3.5 µm. The raphe are straight with the proximal ends dilated slightly and the distal ends are deflected onto the valve mantle. The axial area is narrow but expands near the central area. The isolated pores are absent. The striae are short and radiate 11–12 in 10 µm.

SEM, external view (Figure 12F,G). The striae are uniseriate and not interrupted near the valve face–mantle junction and continue in uniseriate rows onto the mantle. The areolae are occluded by large *c*-like semilunar siliceous flaps and merged with the areolar surface, ≈50 in 10 µm. The proximal raphe endings are expanded, pore-like, and deflected towards the isolated round pore. The apical pore field is composed of round porelli and is located along one mantle and the valve terminus.

SEM, internal view (Figure 12H). The areolar opening is small, round, and located in a shallow foraminal row. The side walls of the areolae bear small struts. The proximal raphe ends are hooked and located on a raised central nodule. The pseudosepta are poorly defined and visible at both poles. The helictoglossae are offset from the raphe branch.

**Comments.** *Gomphonema trifonovae* sp. nov. has a valve shape similar to *G. angustivalva* Reichardt (Table 3). The two species have similar valve widths (2.8–3.5 µm in *G. trifonovae* sp. nov. versus 2.5–3.7 µm in *G. angustivalva*). The two species differ in their densities of striae: 11–12 in 10 µm in *G. trifonovae* sp. nov. versus 14–18 in 10 µm in *G. angustivalva*. *G. trifonovae* sp. nov. has asymmetric valves along the longitudinal axis (Table 3), while in *G. angustivalva*, the valves are symmetrical along the longitudinal axis. The central area in *G. trifonovae* sp. nov. is not pronounced, while in *G. angustivalva*, it is transversely rectangular [54,55,56]. In *G. trifonovae* sp. nov. the isolated pore is absent (Figure 12G,H); in *G. angustivalva*, the external opening of the isolated pore is clearly visible [54,56].

**Figure 13:**
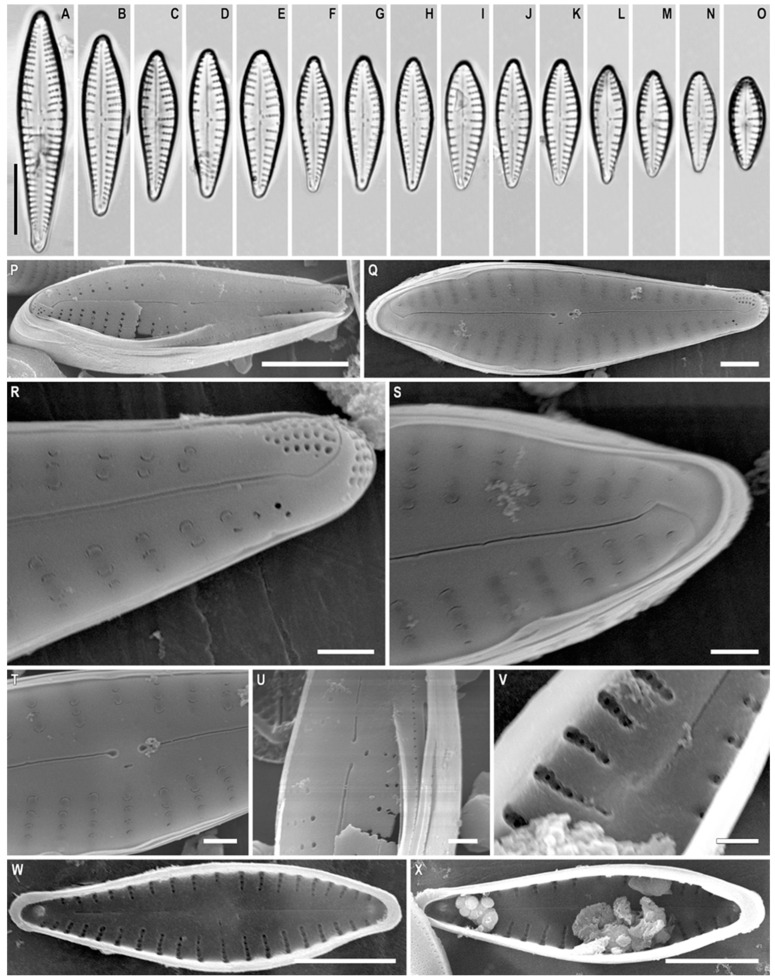
(**A**–**O**). *Gomphonema zapitaja* Kulikovskiy, Kociolek, Solak and *Glushchenko* sp. nov. (**A**–**O**). Light microscopy, differential interference contrast, size diminution series. Slide no. 18599 (**A**–**E**,**G**,**H**,**J**–**O**) and 18606 (**F**,**I**). (**P**–**U**). Scanning electron microscopy, external views. (**V**–**X**). Scanning electron microscopy, internal view. (**B**). Holotype. Scale bar (**A**–**O**) = 10 μm; (**P**,**W**,**X**) = 5 μm; (**Q**) = 2 μm; (**R**–**V**) = 1 μm.

*Gomphonema zapitaja* Kulikovskiy, Kociolek, Solak and *Glushchenko* sp. nov. (Figure 13)

**Holotype.** Deposited in the herbarium of MHA, Main Botanical Garden Russian Academy of Science, Moscow, Russia, holotype here designated, slide no. 18599 (Figure 13B).

**Isotype.** Collection of Maxim Kulikovskiy at the Herbarium of the Institute of Plant Physiology of the Russian Academy of Science, Moscow, Russia, slide no. 18599a.

**Type locality.** Russia, Lake Baikal, shallow-water bay, fragments of filamentous algae and higher plants, 52°27.042′ N; 106°53.215′ E. Collected by M.S. Kulikovskiy, 14 July 2011, pH = 9.3, conductivity = 151 µS cm^−1^, t = 21.3 °C.

**Etymology.** The word “*zapitaja*” in Russian means comma and the epithet refers to the shape of the valves.

**Distribution.** Known only from the type locality.

**Description.** LM (Figure 13A–O). The valves are clavate and are asymmetrical to the longitudinal axis with an acutely rounded headpole. The footpole is narrowly rounded. The valve length is 13.5–34.5 µm and the breadth is 5–7 µm. The raphe is straight with the proximal ends dilated slightly and the distal ends deflected onto the valve mantle. The axial area is narrow but expands near the central area. The central area is formed by a shortening of the central striae. There is an isolated pore near the central nodule and is densely spaced to the rather long median stria. The striae are short and radiate at 11–15 in 10 µm. The striae radiate at 11–13 in 10 µm.

SEM, external view (Figure 13P–U). The striae are uniseriate. The areolae are occluded by large *c*-like semilunar siliceous flaps and merged with the areolar surface, ≈40 in 10 µm. The proximal raphe endings are expanded, pore-like, and deflected towards the isolated round pore. The apical pore field is composed of round porelli and is asymmetrical between the two sides of the footpole. The distal raphe end is strongly hooked to bisect the apical pore field.

SEM, internal view (Figure 13V–X). The areolar openings are small, round, and located in shallow foraminal rows. The side walls of the areolae bear parts of small struts. The isolated pore is small, transversely elongated, and located in a long groove. The proximal raphe ends are hooked and located on a raised central nodule. The pseudosepta are present, visible at both poles. The helictoglossae offset from the raphe branch.

**Comments.** On the basis of valve shape, *Gomphonema zapitaja* sp. nov. is similar to *G. cymbelliclinum* and *G. angustatum* (Table 4). The valves of *G. zapitaja* sp. nov., similar to the valves of *G. cymbelliclinum*, are asymmetric along the longitudinal axis ([55]; 61, Pl. 40, Figures 1–31). *G. zapitaja* sp. nov. differs from G*. cymbelliclinum* ([57], Pl. 40, Figures 1–31) in having more acute headpole and footpole of the valves.

The valves of *G. zapitaja* sp. nov. are more club shaped and the footpole is more tapered than the headpole (Figure 13A–O); these features are less evident in G*. cymbelliclinum*. The internal opening of the isolated pore in *G. zapitaja* sp. nov. (Figure 13V,W), as in *G. cymbelliclinum*, is slit-like ([57], Pl. 40, Figure 33). At the same time, the slit of the isolated pore of *G. zapitaja* sp. nov. is shorter. The striae in *G. zapitaja* sp. nov. consist of areolae covered with siliceous flaps, while in *G. cymbelliclinum* the areolae are not covered ([55], Pl. 90, Figure 3; 61, Pl. 40, Figure 33). Pore fields of *G. zapitaja* sp. nov. are formed by large poroids, asymmetrically developed, and separated by the distal raphe branch. One part of the pore field extends onto the front surface of the valve and the second part is predominantly located on the valve margin (Figure 13R). In *G. cymbelliclinum*, the pore field is formed by smaller poroids; the poroids almost do not extend onto the front surface of the valve ([55], Pl. 90, Figure 6).

*G. zapitaja* sp. nov. resembles *G. angustatum* in valve shape. The species have similar valve widths (5–7 µm in *G. zapitaja* sp. nov. and 5.3–6.7 µm in *G. angustatum*) and striae densities (11–13 µm in *G. zapitaja* sp. nov. and 10–14 µm in *G. angustatum*). Both species have areolae covered with siliceous flaps ([55], Pl. 138, Figures 1 and 4). In both species, the poroids of the pore fields extend onto the front surface of the valves (Table 4; ref. [55], Pl. 138, 1). However, *G. zapitaja* sp. nov. has asymmetry of the valves along the apical axis, which easily distinguishes it from *G. angustatum* (Table 4).

**Figure 14:**
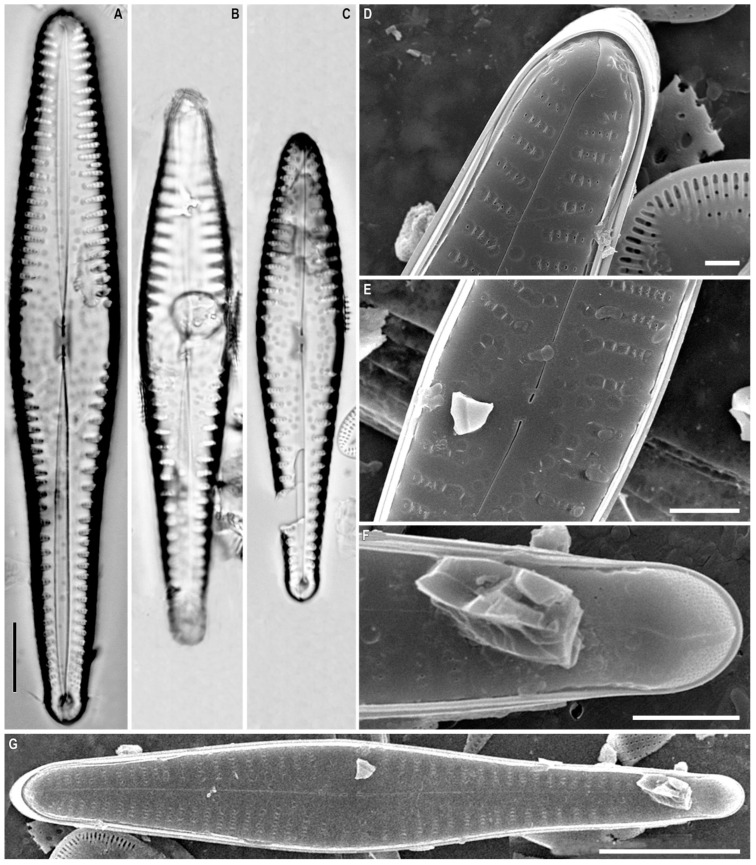
(**A**–**G**). *Gomphonema megabaicalensis* Kulikovskiy, Kociolek, Solak and *Glushchenko* sp. nov. (**A**–**C**). Light microscopy, differential interference contrast, size diminution series. Slide no. 18609. (**D**–**G**). Scanning electron microscopy, external views. (**A**). Holotype. Scale bar (**G**) = 20 μm; (**A**–**C**) = 10 μm; (**E**,**F**) = 5 μm; (**D**) = 2 μm.

**Figure 15:**
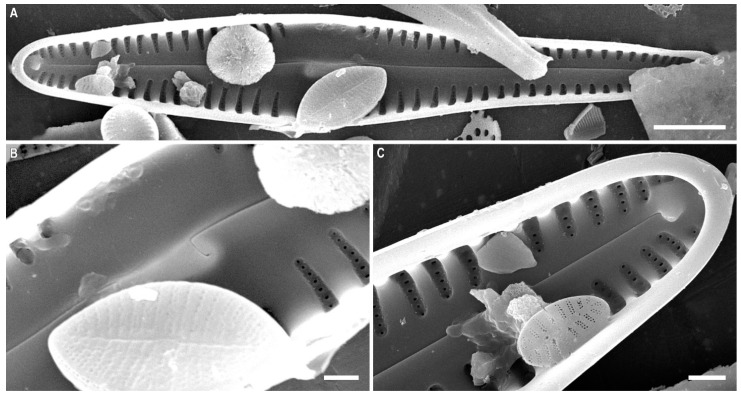
(**A**–**C**). *Gomphonema megabaicalensis* Kulikovskiy, Kociolek, Solak and *Glushchenko* sp. nov. Scanning electron microscopy, internal views. Scale bar (**A**) = 10 μm; (**B**,**C**) = 2 μm.

*Gomphonema megabaicalensis* Kulikovskiy, Kociolek, Solak and *Glushchenko* sp. nov. (Figure 14A–G and Figure 15A–C)

**Holotype.** Deposited in the herbarium of MHA, Main Botanical Garden Russian Academy of Science, Moscow, Russia, holotype here designated, slide no. 18609 (Figure 14A).

**Isotype.** Collection of Maxim Kulikovskiy at the Herbarium of the Institute of Plant Physiology Russian Academy of Science, Moscow, Russia, slide no. 18609a.

**Type locality.** Russia, Lake Baikal, shallow-water bay, bottom sediments, 52°27.042′ N; 106°53.215′ E. Collected by M.S. Kulikovskiy, 14 July 2011, pH = 7.5, conductivity = 295 µS cm^−1^, t = 25.0 °C.

**Etymology.** The epithet refers to the type locality and the large-sized specimens.

**Distribution.** Known only from the type locality.

**Description.** LM (Figure 14A–C). The valves are heteropolar, clavate with broadly rounded or subclavate headpoles, and widely rounded with weakly protracted footpoles. The valve length is 68–103 µm and the breadth is 12.5–16.0 µm. The raphe is lateral with the proximal ends dilated slightly and the distal ends deflected onto the valve mantle. The axial area is wide and gradually widens towards a rectangular central area. The isolated pore is absent. The striae weakly radiate at 5–7 in 10 µm.

SEM, external view (Figure 14D–G). The striae are uniseriate. The areolae are rounded or elongated and located in small round depressions, separated by a thickened rim, ≈20 in 10 µm. The proximal raphe endings are expanded, pore-like, and deflected towards the isolated round pore. The apical pore field bilobed is composed of round porelli.

SEM, internal view (Figure 15A–C). The areolar openings are large and round without internal occlusions and located in deep foraminal rows between the strongly silicified vimines. Beneath the vimines, the sides of the grooves bear pairs of small struts. The proximal raphe ends are hooked and located on a raised central nodule. The pseudosepta are present and visible at the headpole. The helictoglossae are offset from the raphe branch.

**Comments.** *Gomphonema megabaicalensis* sp. nov. has a large axial and central area bearing various depressions. However, the new species is well distinguished from similar species (Table 5). *G. megabaicalensis* sp. nov. differs from *G. oxycephalum* P.T. Cleve in the club-shaped valves and *G. oxycephalum* has rhomboid-lanceolate club-shaped valves. The valves of *G. megabaicalensis* sp. nov. are noticeably narrower than *G. oxycephalum* (12.5–16.0 in *G. megabaicalensis* sp. nov. versus 18–23 µm in *G. oxycephalum*). *G. megabaicalensis* sp. nov. has a rectangular central area, defined by a few small, shortened striae, or in the form of a fascia.

The striae in the central area of the valves of the species *G. oxycephalum* are not shortened; the axial area expands and passes into the central area ([52], Pl. 147, Figures 1–4). The two species also have ultrastructural differences.

*G. megabaicalensis* sp. nov. has small maculae on the external surface of the valves and small thickenings between the areolae (Figure 14D,E). In *G. oxycephalum* the inlay of the surface of the valves is more complex ([52], Pl. 148, Figures 1–4). In *G. megabaicalensis* sp. nov., an isolated pore is absent (Figure 14E and Figure 15A,B).

*G. megabaicalensis* sp. nov. has some similarities with *G. demerarae*: these species lack isolated pores, have areolae with a crater-like structure, and possess chaotically located maculae present on the front surface of the valves ([52], Pl. 152, Figures 1–5; Pl. 153, Figures 1 and 4). However, in *G. megabaicalensis* sp. nov. the striae are always uniseriate, with crater-shaped areolae lying in individually-rounded depressions which are separated by small thickenings (Figure 14D,E). In *G. demerarae*, the striae consist of two rows of crater-shaped or slit-like areolae without any thickenings, and the areolae of the striae are located in small individual or general depressions ([52], Pl. 153, Figures 2–5). *G. megabaicalensis* sp. nov. also differs from *G. demerarae* in having more narrow valves in the central part and the less tapering headpole of the valves ([52], Pl. 152, Figures 1–5). *G. megabaicalensis* sp. nov. is noticeably larger than *G. demerarae*, and in terms of the width of the axial area, *G. megabaicalensis* sp. nov. has a smaller axial area than *G. demerarae* ([52], Pl. 152, Figures 1–5). Striae density in *G. megabaicalensis* sp. nov. is lower (5–7 in 10 µm) than in *G. demerarae* (8–9 in 10 µm).

*G. megabaicalensis* sp. nov., similar to *G. spectabilissimum* Metzeltin and Lange-Bertalot 1998, has maculae ([52], Pl. 146, Figures 1–3, Pl. 162, Figures 3–5). At the same time, *G. megabaicalensis* sp. nov. has club-shaped valves, while in *G. spectabilissimum* the valves are linear, being only slightly club shaped*. G. megabaicalensis* sp. nov. has a well-defined central area (Figure 14E), while in *G. spectabilissimum* the central area is lacking ([52], Pl. 146, Figures 1–3, Pl. 162, Figures 3 and 4). Even more differences are found in the ultrastructure of these two species. In the *G. megabaicalensis* sp. nov. are striae formed from 3–8 areolae, while those of *G. spectabilissimum* are shorter, formed from 2–4 areolae. The structure of the areolae also differs between the two species: in *G. megabaicalensis* sp. nov., the areolae are crater-shaped, lying in individual depressions separated by small thickenings, while in *G. spectabilissimum* the areolae of the stria lie in a common depression, and each of the areolae is covered with a siliceous flap ([52], Pl. 162, Figures 3–5). There are also differences in the structure of the raphe: in *G. megabaicalensis* sp. nov., the central raphe ends are simple and slightly widened, while in *G. spectabilissimum*, the central raphe ends lie in widening depressions ([52], Pl. 162, Figure 3). Silica granules are present at the boundary between the front surface and valve inflection in *G. spectabilissimum*, which are absent in *G. megabaicalensis* sp. nov. In *G. megabaicalensis* sp. nov. the isolated pore is absent (Figure 14E and Figure 15A,B), while in *G. spectabilissimum* the external isolated pore opening is clearly seen in SEM ([52], Pl. 162, Figures 3 and 4).

The data presented herein, along with other published observations, are beginning to help develop an understanding of the distribution species and genera of diatoms within the Lake Baikal and Transbaikal region, as well as the variety of morphological groups that inhabit this amazing freshwater ecosystem. Distributional patterns may be viewed spatially and temporally in the lake and region.

Both large-scale sampling surveys [20,58] and more focused collections (the majority of published results) have shown that within Lake Baikal, species can be found in certain basins or regions. Due to rifts within the Baikal basin, the lake can be split latitudinally into the northern, central, and southern basins, each with its own physical and chemical composition [1]. The northern basin, which has deep waters, is also the least developed in terms of anthropogenic impacts and harbors many endemic diatom genera and species in the lake, including species of *Gomphonema* [10,20,26]. The central basin, which is the easiest access point to the lake due to the geographic position relative to the communities of Irktusk and Lystvyanka, has had many species described exclusively from this region, including gomphonemoid diatoms [13,14,25,29,33]. The southern basin, characterized in general as being more shallow and, due to the inputs from the Selenga River delta from the eastern side of the lake, has been shown to have its own floristic composition [59,60]. In this latter region, anthropogenic nutrient inputs have changed the cycling of silica and the diatom flora [61].

In addition to the north–south axis of the lake, a spatial difference in diatoms also occurs with respect to the east and west shores of Lake Baikal. Of course, the strongest gradient in this regard is driven by the inputs of the Selenga River (as described above), the data presented herein also suggest some differences in the diatom flora between the eastern and western shores. For instance, all of the new species described herein are known only from the eastern shore of Lake Baikal, while previous considerations of the *Gomphonema* species collected from the western shore were not encountered in the current study. As indicated, many studies originated in Lake Baikal in the central basin of the western shore due to logistical reasons. The smaller number of taxa described from the eastern shore is likely due in large part to sampling intensity since most work on Lake Baikal has originated and focused on collections from the western shore.

Among the representatives of the genus *Gomphonema* found in Lake Baikal, there are many that appear to be endemic [13,14,25,34,62,63], though there are also species found in the lake that are more widely distributed [25]. In the present report, we note species that have been found beyond Lake Baikal, especially in Siberia. These include species such as *Gomphonema makarovae*, *G. distans*, *G. subarcticum*, *G. parvulius*, *G. duplipunctatum*, *G. sphenovertex*, *G. jergackianum*, *G. popovae*, *G. medioasiae*, *G. demersum,* and *G. auguriosiberica*. These species were described previously from Siberia and the Arctic zone of Eurasia. *Gomponema makarovae*, for example, was described from a small, unnamed freshwater lake near the White Sea, Arkhangelsk Oblast, Russia [64]. The report of this species from Lake Kanas in China [65] requires verification. *Gomphonema subarcticum* was described from a *Sphagnum* collection from the Yugorskiy Shar Peninsula, NW Siberia, Russia [64]. Later it was found in Lake Elgygytgyn, Chukotka, Russia [66]. It is a circumboreal oligotrophic and dystrophic species. *Gomphonema distans* was described from Finnish Lapland as *G. lagerheimii* var. *distans* [67]. The taxon was later discovered in Siberia and a taxonomic combination was proposed by the authors [64]. The species is distributed in the northern regions of the Holarctic [68]. *Gomphonema demersum* is probably widespread in Siberia [44]. According to Reichardt [44], *G. demersum* is a complex of closely related species with similar morphology. *Gomphonema parvulius* is known from northern and central Europe and is characterized as an oligotrophic species [54,55]. *Gomphonema duplipunctatum* has been described in Finland. It is known from Europe [55] and the Northwestern USA [69]. Ecologically it is characterized as an oligotrophic-mesotrophic Holarctic species [55,68]. *Gomphonema sphenovertex* was described in Finland and has also been reported in Scandinavia and Iceland [70]. This is a rare species that does not have large populations. *Gomphonema jergackianum* was described from the Western Sajan Mountains, Russia [44]. An oligotrophic species, it is also known to be from Finland, Iceland, and Germany [44,70]. *Gomphonema popovae* is known to be from Russia, Mongolia, and China. *Gomphonema medioasiae* is known to be from Mongolia [50]. The species was previously known only from its type locality, the Barchuluut River [50]. The species found in Baikal and in Siberia may represent a group of species described by Vereshchagin [71] as a Siberian–Baikal species, representing an intermediate group of taxa that may be endemic to either Lake Baikal or Siberia. These observations suggest that benthic diatoms (as opposed to the plankton flora [72] in Lake Baikal and the surrounding Transbaikal region may follow distribution patterns seen in elements of the Baikal fauna [45]. Further research is needed to explore the distributional patterns of benthic diatoms in Lake Baikal and their relationships to species exclusively endemic to each region. *Gomphonema pseudoaugur* is known from the Holarctic [54]. An oligotrophic species, it is often observed in meso- and eutrophic lakes [54].

The *Gomphonema* diatom flora of Lake Baikal is rich in terms of the total number of taxa though it is also diverse in terms of the presence of morphologically distinct groups. In terms of richness, for both endemic and more widely distributed taxa, there have been over 100 species reported from this single lake. Unlike the situation in the closely related family Cymbellaceae, which less than four decades ago was recognized as a single genus [73,74] and now has been split up into almost 20 genera [75,76,77,78,79], the morphological groups within *Gomphonema* [80] have rarely been separated out as separate genera (Lange-Bertalot 1995; Kociolek et al. 2015). Despite this, we can distinguish numerous morphological groups in the genus, many of which are found in Lake Baikal. Morphological groups of the *Gomphonema* species present in Lake Baikal include members of the “classical” species of the genus [25] and the *G. auguriosiberica* sp. nov. described herein. Groups with c-shaped areolae, similar to the classical group, vary in some other features (robust valves such as in *G. popovae*; isolated pore connected to the striae as in *G. baicaldermersus* sp. nov.; astigmate taxa such as *G. genkalii* sp. nov.) are also present. Other species groups previously recognized, but not included in the present treatment, include the *G. ventricosum* group (with a special type of occlusion in the areolae [34,75,80]; and very large, robust species [13] which may have close allies elsewhere in Asia [81] and species with doubly punctate striae. Two species described herein that require further attention are G. *trifonovae* sp. nov. and *G. zapitaja* sp. nov. These species have cells that are both asymmetrical to the apical and transapical axes and, unlike other genera with that combination of symmetry features (e.g., *Afrocymbella* Krammer, *Gomphocymbellopsis* Krammer) [76], these small species have their bilobed apical pore fields (APF) positioned asymmetrically on the footpole, with one lobe along one margin and the other positioned at the base of the footpole. This unique APF construction is found nowhere else amongst the gomphonemoid diatoms (let alone within *Gomphonema* specifically), warranting observations of their growth habit and systematic position.

## 3. Materials and Methods

**Sampling.** Samples used in this publication were collected by Maxim Kulikovskiy in 2011 from a shallow-water bay from the south part of Lake Baikal at 8 km from Enkhaluk village or small pools near it (near Oimur, Kabansky District) located on the eastern shore of Lake Baikal. Physical and chemical water parameters were measured with a Hanna Combo (HI 98129) multiparameter probe (Hanna Instruments, Inc., Woonsocket, RI, USA). Samples were collected from different parts of the bay and from different substratum. A list of slides and their characteristics are given see Table 6.

**Preparation of slides and microscope investigation**. Samples were processed by means of a standard procedure involving treatment with 10% HCl and concentrated hydrogen peroxide. After treatment with HCl, the samples were washed with deionized water. Permanent diatom preparations were mounted in Naphrax^®^ (Brunel Microscopes, Chippenham, UK). Light microscopic (LM) observations were performed with a Zeiss Axio Scope A1 (Zeiss, Oberkochen, Germany) microscope equipped with an oil immersion objective (x100/n.a.1.4, DIC). Valve ultrastructure was examined by means of a JSM-6510LV scanning electron microscope (JEOL Ltd., Akishima, Tokyo, Japan), operated at 10 kV and 11 mm distance. For scanning electron microscopy (SEM), parts of the suspensions were fixed on aluminum stubs after air drying. The stubs were sputter coated with 50 nm of gold in an Eiko IB 3 (Eiko Engineering, Yamazaki, Hitachinaka Shi, Ibaraki Ken, Japan).

Samples and slides are deposited in the collection of Maxim Kulikovskiy at the Herbarium of the Institute of Plant Physiology Russian Academy of Sciences, Moscow, Russia.

## Figures and Tables

**Table 1:** Comparison of the morphological features of some finds of *G. popovae*.

	*G. popovae*	*G. popovae* (as *G. liyanlingae*)	*G. popovae* Morphotype 1	*G. popovae* Morphotype 2
**References**	[48]	[44,50]	This study	This study
**Outline**	heteropolar, lanceolate-clavate	linear-clavate	heteropolar, lanceolate-clavate	heteropolar, lanceolate-clavate
**Headpole**	widely rounded	broadly rounded	broadly rounded	broadly rounded
**Footpole**	narrowly rounded	obtusely rounded	acutely rounded	acutely rounded footpole
**Axial area**	narrow, sometimes extended towards the central part	narrow in smaller species becoming moderately wider in larger ones	narrow, sometimes extended towards the central part	narrow, sometimes extended towards the central part
**Central area**	wide, transversely expanded, sometimes slightly rhombic, bordered in the central part of the valve, usually by one or more irregularly shortened striae on each side	shaped roughly rhombical proximally, then abruptly expanded to a broad fascia at either side with one regularly shortened stria pair in the middle of a few additional irregularly shortened striae	wide, transversely expanded, or slightly rhombic, bordered by 1–3 shorted striae	wide, transversely expanded, or slightly rhombic, bordered by 1–3 shorted striae
**Valve length (μm)**	47–94	42–100	22.5–79.0	43.5–67.0
**Valve breadth (μm)**	8.0–13.3	10–16	6.5–12.0	8.0–9.5
**Striae in 10 μm**	9.5–11.5 at the head part, 9.5–11.0 at the basal part	9–10	9–13	11–13
**Areolae in 10 μm**	15–16 (18–20) *	15–17	18–20	18–20
**Distribution**	Russia, Mongolia, China	Mongolia	Russia, Baikal	Russia, Baikal

* counted from published data.

**Table 2:** Comparison of morphological features of *Gomphonema baicalodemersum* sp. nov. and related species.

	*G. baicalodemersum* sp. nov.	*G. medioasiae*	*G. demersum*
**Outline**	slightly heteropolar, rhombic lanceolate (in larger specimens), or very slightly clavate (in smaller specimens)	only very slightly clavate but almost naviculoid symmetrical to the transapical axis since head poles appear weakly broader than basal poles and lateral margins gradually taper to the poles	lanceolate, only very slightly gomphonemoid-cuneate
**Headpole**	acutely rounded	acutely rounded	obtusely rounded
**Footpole**	acutely rounded	acutely rounded	narrower than headpole
**Axial area**	lanceolate, widening at the central area	barely or not separated appearing lanceolate	lanceolate, widening at the central area
**Central area**	weakly expressed	barely or not separated appearing lanceolate	broad rhombic lanceolate
**Valve length (μm)**	14.5–38.5	47–52	13–41
**Valve breadth (μm)**	3–6	6–8	3.6–6.0
**Striae number in 10 μm**	11–15	8–11	12–16
**Areolae number in 10 μm**	≈30	16–18	≈30 *
**Isolated pore type**	externally, the circular opening located near the central nodule; internally, large, transversely elongated, and located in a long groove, turning into foraminal rows	located near the central nodule and densely spaced to the rather long median stria	the circular external stigma opens internally in an elongated pore
**Distribution**	Russia. Baikal	Mongolia	Russia. Baikal
**References**	This study	[50]	[44]

* Counted from published data.

**Table 3:** Comparison of morphological features of *Gomphonema trifonovae* sp. nov. and related species.

	*G. trifonovae* sp. nov.	*G. angustivalva*
**Outline**	clavate, asymmetrical to the longitudinal axis	slightly heteropolar, linear to linear lanceolate
**Headpole**	acutely to narrowly rounded	narrowly rounded
**Footpole**	acutely rounded	narrowly rounded
**Axial area**	narrow but expands near the central area	narrow, linear
**Central area**	is an extension of the axial area	large, transversely elongated
**Valve length (μm)**	13–26	14–34
**Valve breadth (μm)**	2.8–3.5	2.5–3.5
**Striae number in 10 μm,**	11–12	15–18
**Areolae type**	*c*-like	*c*-like
**Isolated pore**	absent	present
**Distribution**	Russia, Baikal	Europe
**References**	This study	[54,55,56]

**Table 4:** Comparison of morphological features of *Gomphonema zapitaja* sp. nov. and related species.

	*G. zapitaja* sp. nov.	*G. cymbelliclinum*	*G. angustatum*
**Outline**	clavate, asymmetrical to the longitudinal axis	slightly heteropolar, linear to linear-lanceolate	lanceolate to rhombic lanceolate, larger specimens less club-shaped than the smaller
**Headpole**	acutely rounded	rostrate to subcapitate	wedge-shaped, blunter to more pointed
**Footpole**	narrowly rounded	rostrate	barely narrower in larger specimens
**Axial area**	narrow but expands near the central area	very narrow, linear	narrowly linear
**Central area**	formed by shortening of the central striae	large, asymmetric, wider on the side opposite to the isolated pore, bordered on each margin by a single shortened stria	usually extending over more than half of the valve by strong unilateral shortening of the central striae
**Valve length (μm)**	13.5–34.5	16.0–37.5	16–48
**Valve breadth (μm)**	5–7	4.5–6.7	5.3–6.7
**Striae number in 10 μm,**	11–13	9–16	10–14
**Areolae type**	occluded by large *c*-like semilunar siliceous flaps, merged with the areolar surface	not occluded	occluded by large *c*-like or reniform semilunar siliceous flaps, merged with the areolar surface
**Areolae number in 10 μm**	≈40	30–35	35–40
**Internal opening of isolated pore**	slit-like	slit-like	slit-like
**Distribution**	Russia, Baikal	Europe	Europe
**References**	This study	[55,57]	[54,55]

**Table 5:** Comparison of morphological features of *Gomphonema baicalodemersum* sp. nov. and related species.

	***G. megabaicalensis*** **sp. nov.**	***G. oxycephalum*** *	***G. demerarae*** *	***G. spectabilissimum***
**Outline**	heteropolar, clavate	rhomboid-lanceolate clavate	rhomboid-lanceolate clavate	linear, weakly clavate
**Apex shape**	broadly rounded or subclavate headpole and widely rounded, weakly protracted footpole	wedge-shaped headpole and widely rounded, protracted footpole	wedge-shaped headpole and widely rounded, protracted footpole	bluntly rounded headpole and footpole
**Axial area**	wide, gradually widening towards central part of valve	wide, gradually widening towards central part of valve	broadly lanceolate, abruptly widening towards central part of valve	wide, gradually widening towards central part of valve
**Central area**	rectangular, in the form of a fascia or bordered to shortened striae	is an extension of the axial area	absent	is an extension of the axial area
**Valve length (μm)**	68–103	65–110	65–125	90–120
**Valve breadth (μm)**	12.5–16.0	18–23	15–20	11–13
**Striae type**	uniseriate	uniseriate	biseriate, very short	uniseriate
**Striae number in 10 μm,**	5–7	6–8	8–9	5
**Areolae type,**	rounded or elongated, located in small round depressions, separated by a thickened rim	rounded or elongated, located in small round depressions, separated by a thickened rim	slit-like or crater-like	*c*-shaped, located in longitudinal depressions
**Isolated pores**	absent	present	absent	present
**Distribution**	Russia, Baikal	South America. Venezuela, Guyana	South America. Brazil, Guyana	Guyana
**References**	This study	[52]	[52]	[52]

* Characterized and counted from published data.

**Table 6:** List of the slides.

Slide No.	Locality	Coordinates	Substratum	Cond., µS/cm	pH	t, °C	Collection of Date
18589	Lake Baikal, pool near shallow-water bay	52°27.042′ N 106°53.215′ E	fouling on a tree submerged in water	151	9.3	21.3	14 July 2011
18599	Lake Baikal, shallow-water bay	52°27.042′ N 106°53.215′ E	fragments of filamentous algae and higher plants	151	9.3	21.3	14 July 2011
18606	Lake Baikal, small pool near shallow-water bay	52°27.042′ N 106°53.215′ E	detritus on sands	151	9.3	21.3	14 July 2011
18607	Lake Baikal, shallow-water bay	52°27.042′ N 106°53.215′ E	*Cladophora* spp. on bottom	151	9.3	21.3	14 July 2011
18608	Lake Baikal, shallow-water bay	52°27.042′ N 106°53.215′ E	bottom sediments and detritus in a clump of *Carex* spp.	456	9.0	18.2	14 July 2011
18609	Lake Baikal, shallow-water bay	52°27.042′ N 106°53.215′ E	bottom sediments	295	7.5	25.0	14 July 2011

## Data Availability

Not applicable.
